# N-Terminal Acetylation of α-Synuclein Slows down Its Aggregation Process and Alters the Morphology of the Resulting Aggregates

**DOI:** 10.1021/acs.biochem.2c00104

**Published:** 2022-08-09

**Authors:** Rosie Bell, Rebecca J. Thrush, Marta Castellana-Cruz, Marc Oeller, Roxine Staats, Aishwarya Nene, Patrick Flagmeier, Catherine K. Xu, Sandeep Satapathy, Celine Galvagnion, Mark R. Wilson, Christopher M. Dobson, Janet R. Kumita, Michele Vendruscolo

**Affiliations:** # Centre for Misfolding Diseases, Department of Chemistry, University of Cambridge, Cambridge CB2 1EW, U.K.; ‡ Department of Drug Design and Pharmacology, Faculty of Health and Medical Sciences, University of Copenhagen, Copenhagen DK-2100, Denmark; § Department of Cell Biology, Blavantik Institute, Harvard Medical School, Boston, Massachusetts 02115, United States; ∥ School of Chemistry and Molecular Bioscience, Molecular Horizons Institute, University of Wollongong, Wollongong, NSW 2522, Australia; ⊥ Department of Pharmacology, University of Cambridge, Cambridge CB2 1PD, U.K.

## Abstract

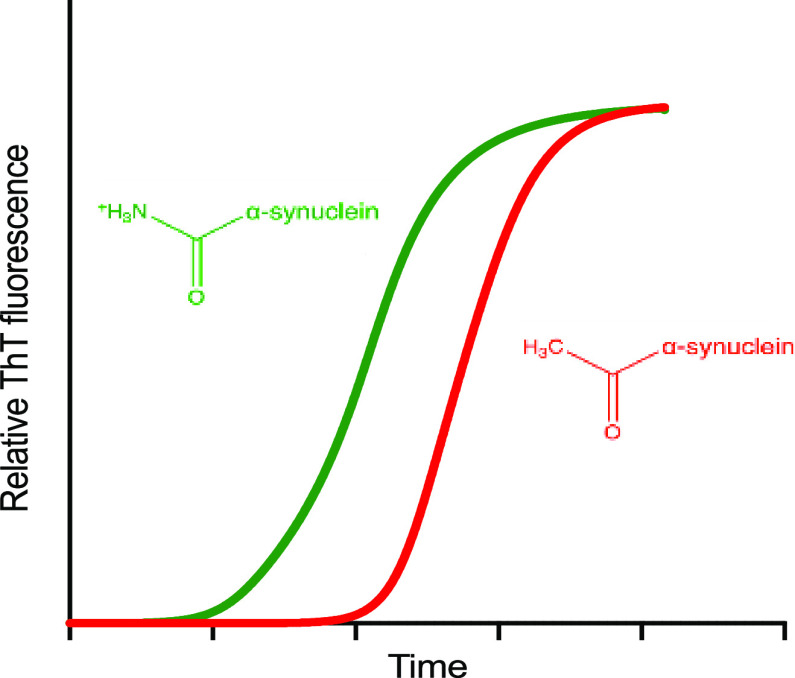

Parkinson’s disease is associated with the aberrant aggregation of α-synuclein. Although the causes of this process are still unclear, post-translational modifications of α-synuclein are likely to play a modulatory role. Since α-synuclein is constitutively N-terminally acetylated, we investigated how this post-translational modification alters the aggregation behavior of this protein. By applying a three-pronged aggregation kinetics approach, we observed that N-terminal acetylation results in a reduced rate of lipid-induced aggregation and slows down both elongation and fibril-catalyzed aggregate proliferation. An analysis of the amyloid fibrils produced by the aggregation process revealed different morphologies for the acetylated and non-acetylated forms in both lipid-induced aggregation and seed-induced aggregation assays. In addition, we found that fibrils formed by acetylated α-synuclein exhibit a lower β-sheet content. These findings indicate that N-terminal acetylation of α-synuclein alters its lipid-dependent aggregation behavior, reduces its rate of in vitro aggregation, and affects the structural properties of its fibrillar aggregates.

## Introduction

Parkinson’s disease (PD) is the second most common neurodegenerative disorder, with over 6 million people worldwide suffering from this condition.^1,2^ Age is a major risk factor of PD, affecting about 1–2% of people over the age of 65 and 4% of people over the age of 85.^3,4^ PD is characterized by a loss of dopaminergic neurons from the *substantia nigra pars compacta* and by the presence of Lewy bodies and Lewy neurites in neurons, which are predominantly composed of aggregates of the 14 kDa intrinsically disordered protein, α-synuclein,^5^ and lipid membranes.^6^

**Figure 1:**
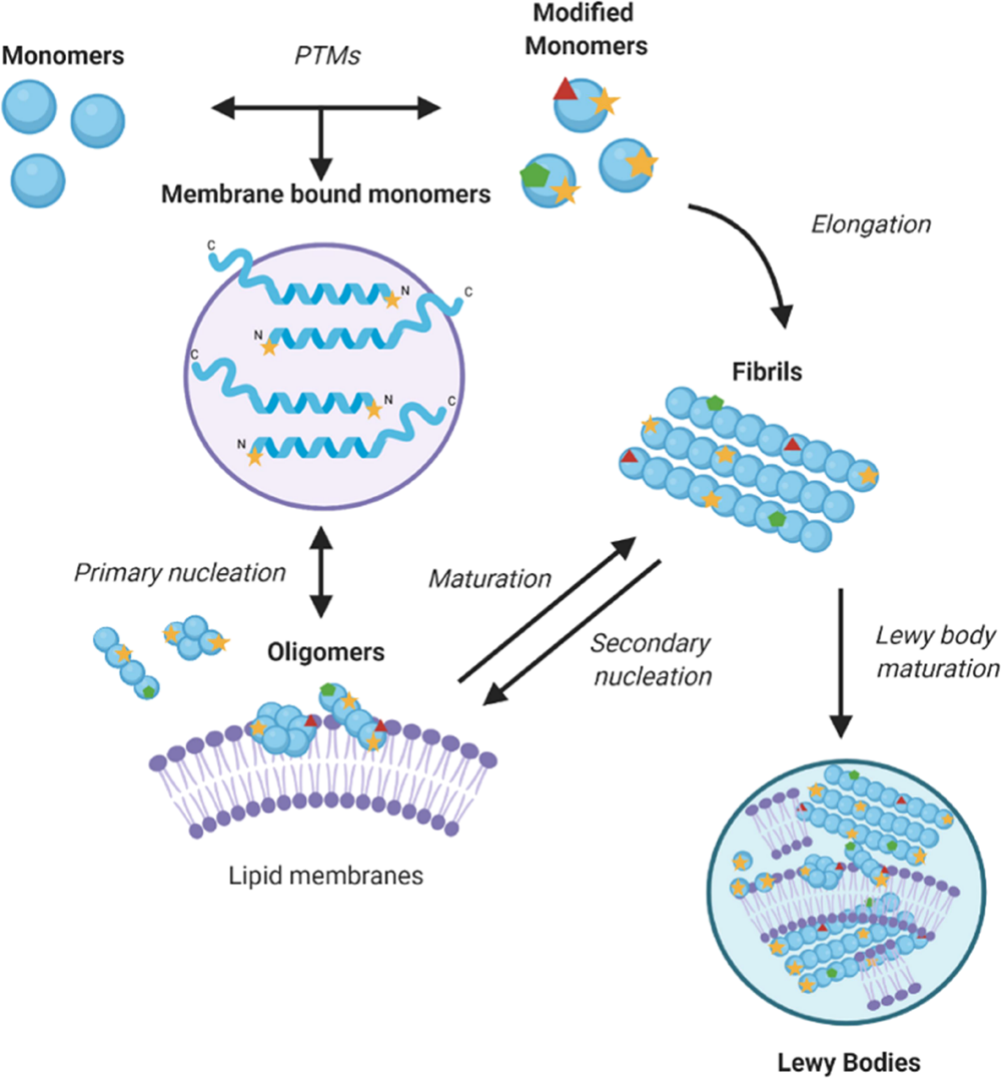
Model of α-synuclein aggregation. As α-synuclein does not readily aggregate spontaneously, it has been proposed that lipid membranes are the site of the primary nucleation events that initiate the process of α-synuclein aggregation in vivo.^71^ At first, monomers nucleate to form small disordered oligomeric species, which then either dissociate and return to the monomeric pool or grow into amyloid aggregates.^17,72^ The process of Lewy body formation may involve such aggregates and lipid membranes.^73,6^

α-Synuclein was linked with PD when *SNCA*, the gene encoding α-synuclein, was found to be mutated in a subset of early-onset PD patients. Six mutations within the *SNCA* gene have been linked with PD (A53T/E, A30P, E46K, H50Q, and G51D); *SNCA* duplication and triplication were also found to cause early-onset familial PD.^7−14^ The functions of α-synuclein involve the regulation of synaptic vesicles sorting, lipid transport, and synaptic plasticity.^15,16^ In neurons, α-synuclein exists primarily as an intrinsically disordered monomer and as a membrane-bound α-helical state.^15,16^ Upon dysregulation, α-synuclein can aggregate to form cross-β amyloid fibrils, via less organized oligomeric intermediates (Figure 1),^17^ which appear to be particularly neurotoxic.^18,19^

**Figure 2:**
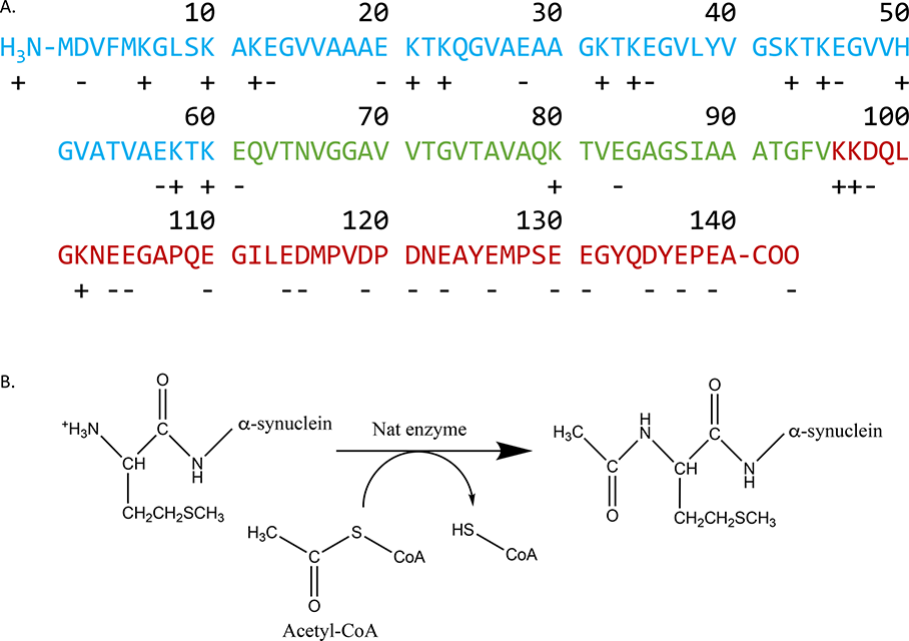
Amino acid sequence of α-synuclein and N-terminal acetylation. (A) Primary sequence of α-synuclein. The N-terminal region (residues 1–60) is shown in blue, the non-amyloid-β component (NAC, residues 61–95) in green, and the negatively charged unstructured C-terminal domain (residues 96–140) in red; positively charged residues are indicated by a + and negatively charged ones by a −. (B) N-terminal acetylation of the −1 Met of α-synuclein. This post-translational modification is carried out by a N-terminal acetyltransferase enzyme. N-terminal acetylation leads to the loss of a positive charge at the N-terminus of α-synuclein.

α-Synuclein is subject to multiple post-translational modifications, including N-terminal acetylation.^20,21^ Cytosolic and pathologically deposited α-synuclein from dementia with Lewy bodies and PD patients, from post-mortem hippocampal, temporal, cingulate, and prefrontal cortical gray matter regions, was analyzed by tryptic digestion and liquid chromatography with tandem mass spectrometry to show that this α-synuclein is constitutively N-terminally acetylated.^22^ N-terminal acetylation is a common post-translational modification carried out in eukaryotes by N-terminal acetyltransferases (Nat) and 85% of eukaryotic proteins are N-terminally acetylated.^23^ To allow for N-terminal acetylation to be introduced in *E. coli*, a pNAT system was developed for the co-expression of a yeast Nat enzyme with the protein of interest.^24,25^ The addition of an acetyl group to the amine group at the N-terminus results in the loss of a positive charge (Figure 2). This change is relevant to the intrinsically disordered monomeric state of α-synuclein, where there are interactions between the negative C-terminal region and positive N-terminal region.^26,27^ Furthermore, the N-terminus is a region of poor solubility^28^ and disrupting the charge or electrostatic interactions may change the biophysical properties of α-synuclein.

In this work, we assessed the effects of N-terminal acetylation on the in vitro aggregation of α-synuclein. We used three conditions, each favoring different individual microscopic processes in the overall aggregation reaction (lipid-induced aggregation, surface-catalyzed fibril amplification, and fibril elongation) to investigate how N-terminal acetylation affected each of these microscopic steps. For each of these conditions, we found that N-terminal acetylation decreased the rate of aggregation. Previous studies have shown that N-terminal acetylation reduces the rate of α-synuclein aggregation in the presence of sodium dodecyl sulfate (SDS) micelles^29^ in non-quiescent conditions^30^ and decreases oligomerization rates in unseeded quiescent conditions.^31^ Other studies reported that N-terminal acetylation increases α-helicity in the N-terminus of α-synuclein and increases its affinity for lipid vesicles.^32−37^ The effects of N-terminal acetylation on the morphology of α-synuclein fibrils has also been studied with varying results. N-terminally acetylated α-synuclein and unmodified α-synuclein have been reported as morphologically indistinct,^32^ or to exhibit differences in fibril periodicity and length.^38^ The stability of both acetylated α-synuclein and unmodified α-synuclein fibrils was reported to be similar in denaturant, but with unmodified α-synuclein being more resistant to protease cleavage.^38^

Under the conditions used in previous studies, it is likely that all microscopic stages of aggregation occur (nucleation, elongation, and surface-catalyzed fibril amplification, Figure 1). In this study, we built on the observation that it is possible to alter the solution conditions to favor the different process of aggregation.^39^ In separating out the stages of aggregation, we can gather more information on the transient aggregation intermediates such as oligomers and protofibrils.^40,41^ Given that α-synuclein is constitutively N-terminally acetylated, we used this approach to investigate how this post-translational modification affects the process of amyloid aggregation, fibril formation, morphology, and stability, particularly as α-synuclein fibrils catalyze the formation of oligomers by surface-catalyzed fibril amplification.^42^

## Results

### N-Terminal Acetylation Does Not Significantly Alter the Conformational Properties of Monomeric α-Synuclein

**Figure 3:**
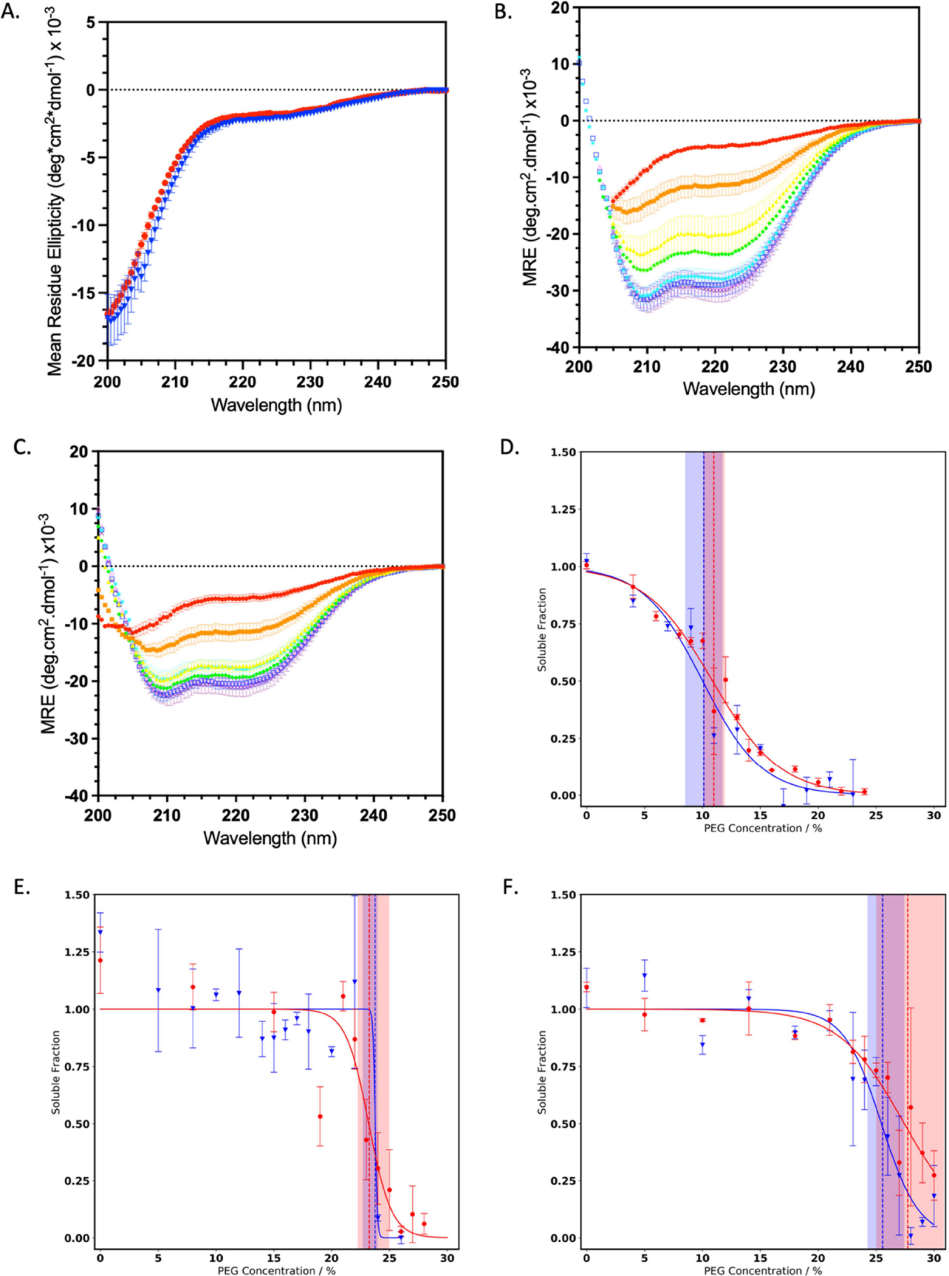
Structural properties and the solubility of monomeric α-synuclein are only slightly affected by N-terminal acetylation. (A) Far UV CD spectra of non-acetylated α-synuclein (blue triangle) and N-terminal acetylated α-synuclein (red dot) monomers; error bars represent the standard error of the mean (SEM) with *n* = 3 (B, C) CD spectra of non-acetylated α-synuclein (B) and acetylated α-synuclein (C), in the presence of increasing concentrations of DMPS: 0.1 mM (red), 0.25 mM (orange), 0.5 mM (yellow), 0.75 mM (green), 1 mM (cyan), 1.5 mM (blue), 2 mM (lilac), and 3 mM (purple); MRE, and error bars represent the SEM with *n* = 3. (D–F) Solubility of monomeric α-synuclein was measured by incubation with increasing concentrations of PEG at pH 4.8 (D), pH 6.5 (E), and pH 7 (F) for non-acetylated α-synuclein (blue) and acetylated α-synuclein (red). The dashed lines represent the PEG_1/2_ value (which is correlated with the solubility) with confidence intervals represented by shaded areas; error bars indicate the standard error of the mean (SEM).

To compare the behavior of N-terminal acetylated and non-acetylated α-synuclein in its monomeric form, the secondary structure was analyzed by circular dichroism (CD) spectroscopy (see the Materials and Methods Section). Both forms of α-synuclein monomers had highly disordered structures as expected (Figure 3A).

Since the ability of α-synuclein to bind to lipid membranes plays an important function role,^16,43^ and lipid membranes may promote α-synuclein aggregation and lead to the presence of lipids in Lewy bodies,^6^ we analyzed the binding of monomeric α-synuclein to vesicles of dimyristoyl phosphatidylserine (DMPS), which have been previously shown to induce the aggregation of α-synuclein.^41^ The binding of α-synuclein to DMPS vesicles was measured by CD spectroscopy (see the Materials and Methods section). We observed slightly less α-helical secondary structure in the protein upon DMPS addition for acetylated α-synuclein (Figure 3C,D). The α-helical content of the N-terminus of the monomeric vesicle bound form of acetylated α-synuclein was reported to increase when bound to SDS vesicles, suggesting that the conformational properties of α-synuclein depend on the composition of the lipid membranes. The change in mean residue ellipticity (MRE) at 222 nm, which reports on the α-helical content, did not show a statistically significant difference in the ability of acetylated and non-acetylated α-synuclein to bind the negatively charged vesicles (Figure 3B–D). The respective dissociation constants (*K*_d_) (Supplementary Methods)^41^ were 5 μM (95% confidence interval 3–10 μM) and 6 μM (95% confidence interval 3–20 μM), respectively, for non-acetylated and acetylated α-synuclein. The stoichiometry of α-synuclein and DMPS molecules was also calculated, where for acetylated α-synuclein, the apparent number of lipid molecules per α-synuclein monomer was lower than that for non-acetylated α-synuclein, with 17 DMPS molecules per acetylated monomer (95% confidence intervals of 10–23) and 29 DMPS molecules per non-acetylated monomer (95% confidence intervals of 22–35, Figure S1). The reduced stoichiometry for acetylated α-synuclein may explain the decreased α-helicity observed compared to the non-acetylated one (Figure 3C,D).

The solubility of monomeric α-synuclein was then analyzed under the conditions used for later kinetic assays (pH 4.8, 6.5, and 7.4), as a decreased solubility of proteins can impact their aggregation propensity.^28^ Acetylated α-synuclein displayed similar behavior to the non-acetylated α-synuclein in the presence of increasing concentrations of polyethylene glycol (PEG) across this pH range, indicating that the solubility of those two species is the same under the conditions that we analyzed (Figure 3E–G).

### N-Terminal Acetylation of α-Synuclein Reduces the Lipid-Induced Aggregation Rate

**Figure 4:**
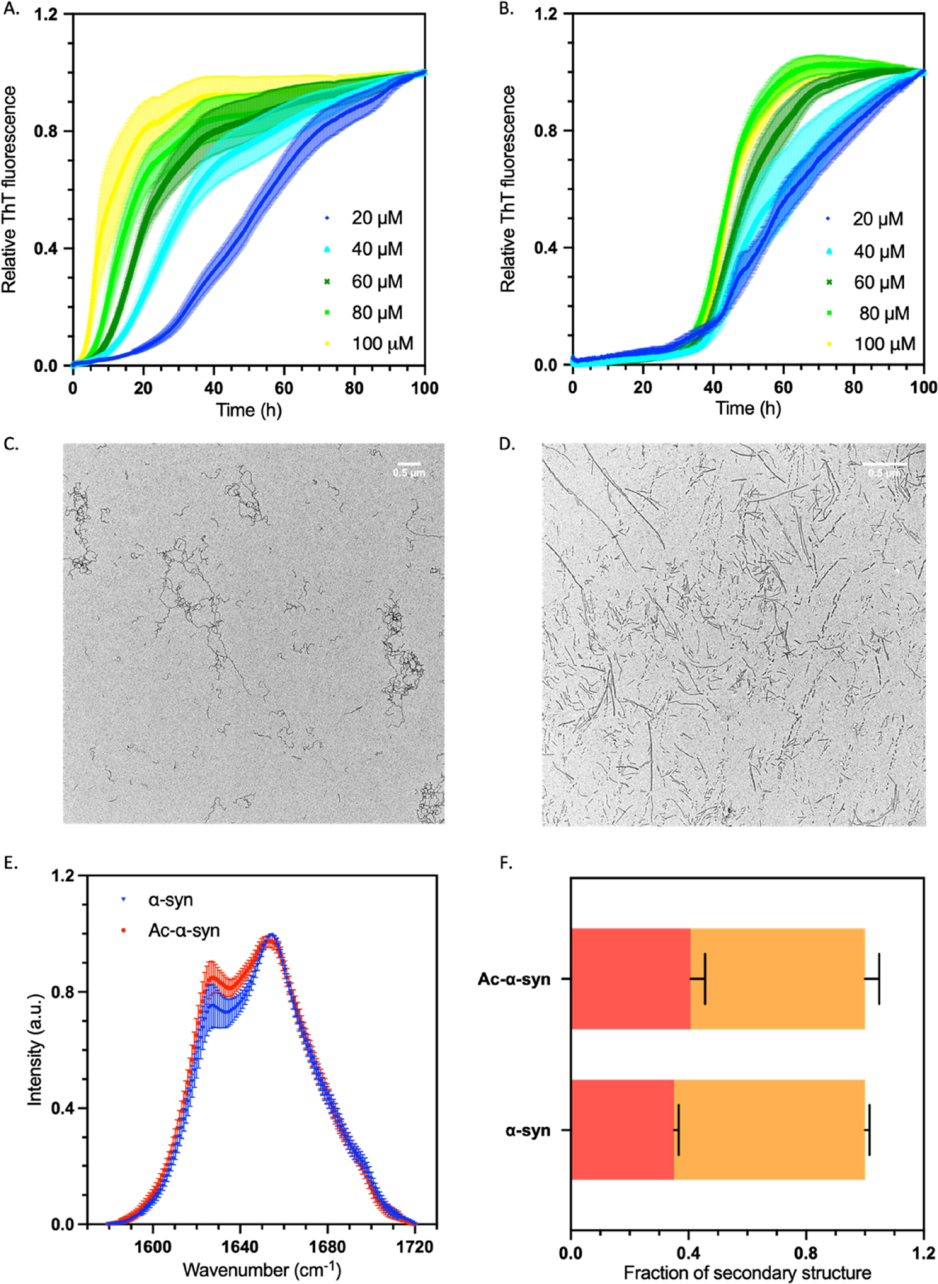
N-terminal acetylation delays the lipid-induced aggregation of α-synuclein. (A, B) Representative time course of a lipid-induced aggregation assay of non-acetylated (A) and acetylated α-synuclein (B); error bars represent the SEM of three repeats. Data were normalized to the end-point ThT fluorescence values for each reaction. Increasing initial concentrations of α-synuclein monomers added to the reaction are shown: 20 μM (dark blue), 40 μM (light blue), 60 μM (dark green), 80 μM (light green), and 100 μM (yellow). Aggregation conditions were as follows: 20 mM NaPO_4_ buffer, pH 6.5, 100 μM DMPS; error bars represent the SEM with n = 3. (C, D) TEM images at 6.5 k magnification of the end point of the aggregation reaction from panels A and B, respectively; the scale bar represents 500 nm. (E) Normalized FTIR spectra of isolated aggregation end products of non-acetylated (blue triangles) and acetylated α-synuclein (red circles); error bars represent the SEM with *n* = 3. (F) Deconvolution of the FTIR spectra into secondary structural content for non-acetylated and acetylated α-synuclein, β-sheet shown in red and α-helix/disordered shown in orange; error bars represent SEM of *n* = 3.

We next investigated the effect of N-terminal acetylation on α-synuclein aggregation in the presence of lipid membranes. To this end, we used negatively charged DMPS vesicles, which were previously shown to induce α-synuclein aggregation.^41^ Lipid-induced aggregation of α-synuclein is mediated by the binding of its positively charged N-terminus (Figure 2) to the negatively charged head groups of DMPS vesicles.^41,44^ Using ThT fluorescence intensity as a measure of fibril mass, we found that the rate of lipid-induced aggregation was decreased for acetylated α-synuclein with respect to non-acetylated α-synuclein (Figure 4A–C). The ThT fluorescence intensity at the end of the aggregation reaction, however, was increased for acetylated α-synuclein compared to non-acetylated α-synuclein (Figure S2), indicating either a higher level of monomer to fibril conversion or the formation of aggregate species with different ThT binding properties.

To further study the structure and morphology of the aggregates formed in the lipid-induced aggregation process, the lipid vesicles were removed by the addition of a detergent. The isolated aggregates were analyzed by Fourier-transform infrared spectroscopy (FTIR) to determine the secondary structure and by transmission electron microscopy (TEM) and atomic force microscopy (AFM) to determine their morphology (Figure 4). At 30 °C, the products of lipid-induced aggregation of non-acetylated α-synuclein are primarily kinetically trapped protofibrils,^41,44,45^ which are characteristically thin (height < 5 nm) and twisted, as we indeed observed by AFM for both acetylated and non-acetylated species (Figure S2), However, when the end products of the lipid-induced aggregation reaction of acetylated α-synuclein were analyzed by TEM, mature fibrils were observed alongside the immature protofibrils, compared to only immature species for non-acetylated α-synuclein (Figure 4C,D). This result was further corroborated by the analysis of the FTIR spectra, which revealed a higher degree of β-sheet structure in the acetylated species (Figure 4E,F). The additional presence of mature fibrils at the end of the acetylated α-synuclein aggregation reaction is likely the cause of the increased ThT fluorescence signal observed in the aggregation of acetylated α-synuclein (Figure S2).

### The N-Terminal Acetylation of α-Synuclein Reduces Fibril Elongation and Surface-Catalyzed Fibril Amplification

After the primary nucleation events to initiate α-synuclein aggregation, fibrils can act as catalysts to seed further aggregation. The seeding by α-synuclein fibrils has been associated with the cell-to-cell spreading of α-synuclein pathology, and previous studies have shown that acetylated α-synuclein is more effective at seeding aggregation in cells.^46^ It has recently been shown that acetylated α-synuclein is recruited to fibrils by interactions involving the N-terminal 11 residues and intrinsically disordered regions of the fibril and oligomer surfaces.^47^ Therefore, we assessed how the N-terminal acetylation of α-synuclein impacted the secondary processes of aggregation by using conditions that favor fibril elongation or surface-catalyzed fibril amplification, which includes both secondary nucleation and fibril elongation steps. By varying solution conditions, we can favor the aggregation processes in our reactions; by using a high concentration of seeds and pH 6.5, fibril elongation is favored, but with a low concentration of seeds and an acidic pH, we can favor secondary nucleation in the surface-catalyzed fibril amplification reaction. Acidity in specific brain regions has been proposed to be important in the development of PD.^48^

**Figure 5:**
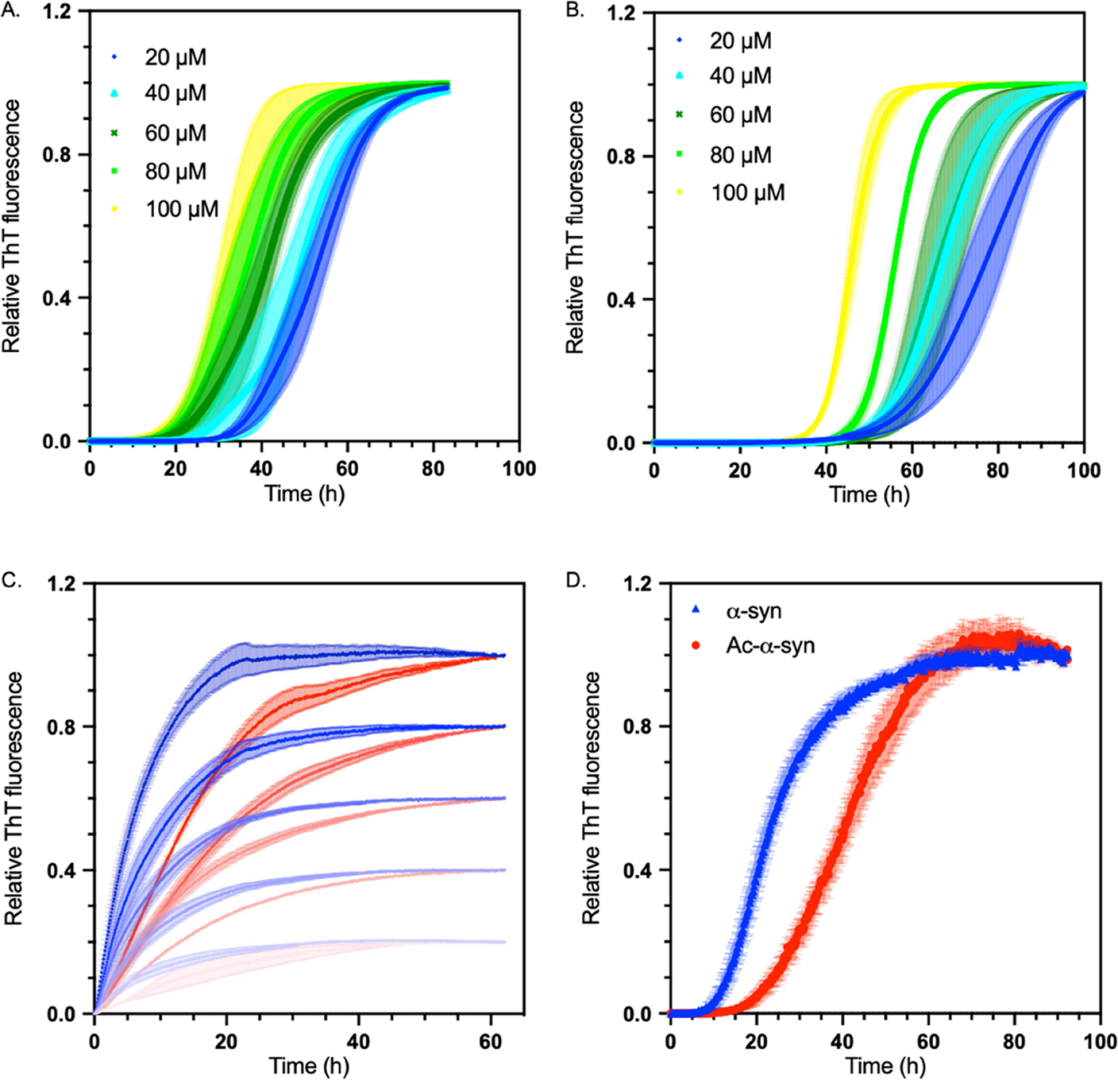
N-terminal acetylation delays secondary processes and fibril elongation of α-synuclein (A) Representative time courses of surface-catalyzed fibril amplification of the non-acetylated α-synuclein monomer seeded by non-acetylated α-synuclein fibrils. (B) Representative time courses of surface-catalyzed fibril amplification of the acetylated α-synuclein monomer seeded by acetylated α-synuclein fibrils. (A, B) Data were normalized to the end-point ThT fluorescence values for each reaction. Increasing initial concentrations of α-synuclein monomers added to the reaction are shown: 20 μM (dark blue), 40 μM (light blue), 60 μM (dark green), 80 μM (light green), and 100 μM (yellow). Aggregation conditions were as follows: 20 mM NaPO_4_ buffer, pH 4.8, 50 nM seeds, under quiescent conditions; error bars represent SEM, over three replicates. (C) Representative time courses of fibril elongation of the non-acetylated α-synuclein monomer seeded with non-acetylated α-synuclein fibril (blue) and acetylated α-synuclein monomer seeded with acetylated α-synuclein fibril (red). Increasing concentrations are represented by increasing color, with 20 μM represented as the lightest and 40, 60, 80, and 100 μM as the darkest. Aggregation conditions were as follows: 20 mM NaPO_4_ buffer, pH 6.5, 2.5 μM seeds, under quiescent conditions at 37 °C. (D) Analysis of aggregation in an unseeded shaking reaction of non-acetylated α-synuclein (blue triangle) and acetylated α-synuclein (red dot); error bars represent the SEM with *n* = 3.

When compared with non-acetylated α-synuclein, acetylated α-synuclein showed reduced rates of both fibril elongation and fibril amplification in relation to non-acetylated α-synuclein (Figure 5). Non-acetylated α-synuclein fibrils have a higher seeding efficiency, suggesting that a small change in the N-terminus that forms part of the fuzzy coat of the fibril surface^49^ may affect both the addition of monomers to fibril ends and the efficiency of surface-catalyzed fibril amplification catalyzed by fibril surfaces.

In 20 mM NaPO_4_ pH 6.5 buffer, under shaking conditions, where it is likely that all microscopic mechanisms occur,^50^ non-acetylated α-synuclein had a higher rate of aggregation compared to acetylated α-synuclein, and a higher ThT fluorescence plateau (Figures 5D, S3A). A decreased ThT plateau signal for aggregated acetylated α-synuclein is consistent with previous studies.^51^ However, in this bulk aggregation assay, where we used the same NaPO_4_ buffer as in the lipid-induced aggregation, the structures of the resulting fibrils did not display significant differences (Figure S3C,D), highlighting the significance of lipids in the aggregation of α-synuclein. When the bulk aggregation was carried out in PBS at pH 7.4, however, acetylated α-synuclein formed fibrils with a different secondary structure (Figure S3), further highlighting the importance of solution conditions and aggregation processes in fibril structure and morphology.

### N-Terminal Acetylation of α-Synuclein Increases the Formation of Misfolded Oligomers

**Figure 6:**
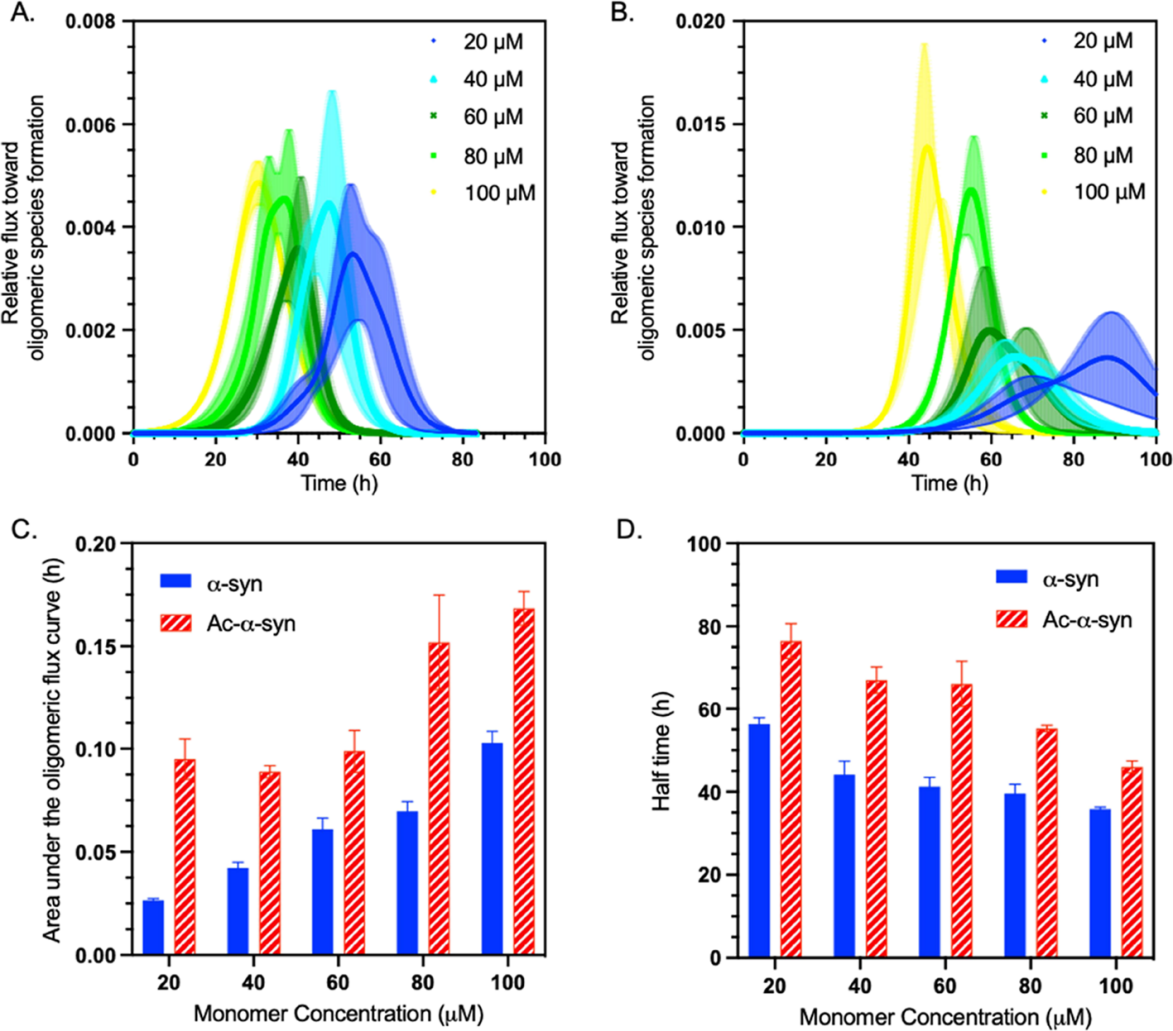
N-terminal acetylation of α-synuclein increases the oligomer populations generated during aggregation. (A, B) Relative flux toward oligomeric species formation (Phi) of non-acetylated (A) and acetylated α-synuclein (B) generated from surface-catalyzed fibril amplification data in Figure 5; error bars represent the SEM with *n* = 3. (C) Area under the curve (Phi AUC) for non-acetylated (blue) and acetylated α-synuclein (red) as a function of monomer concentration; error bars represent the SEM with *n* = 3. (D) Half time of the surface-catalyzed fibril amplification reactions for non-acetylated (blue) and acetylated α-synuclein (red) as a function of monomer concentration; error bars represent the SEM with *n* = 3.

Since α-synuclein oligomers are highly toxic species,^18^ we investigated whether or not N-terminal acetylation affects the formation of oligomeric species in seeded aggregation and the structures of stabilized oligomers. The production of α-synuclein misfolded oligomers during the aggregation process was calculated using a model described recently (see the Methods section in the Supporting Information), harnessing the observed rates of fibril elongation and secondary nucleation to predict the abundance of oligomers in the surface-catalyzed fibril amplification reaction.^40^ We found that the N-terminal acetylation of α-synuclein increases the flux toward misfolded oligomeric species. Although the onset of oligomer production was delayed with an increased lag time, the height of the predicted oligomer peaks was higher in acetylated α-synuclein, indicating a larger concentration of oligomer species over time (Figure 6). As we calculated a difference in oligomer flux, our results indicate that the rate of surface-catalyzed fibril amplification (which includes both secondary nucleation at the fibril surface and fibril elongation at fibril ends) is reduced for acetylated α-synuclein.

In addition to probing the concentrations of oligomers produced during the aggregation process, we also compared the structures of kinetically trapped oligomers of non-acetylated and acetylated α-synuclein, which were produced by a well-established protocol.^52^ Analysis by FTIR indicated no structural differences between these two species, as both displayed similar degrees of β-sheet content, a parameter which has previously been linked with oligomer toxicity, in keeping with previous results^18^ (Figure S4).

### N-Terminal Acetylation Alters the Morphology and Secondary Structure, but Not the Stability, of α-Synuclein Fibrils

In order to obtain more insights into the links between the effects of N-terminal acetylation of α-synuclein on the thermodynamics, kinetics, and cytotoxicity of α-synuclein aggregates, we studied the effects of this post-translational modification on the stability, morphology, and structure of the fibrils.

**Figure 7:**
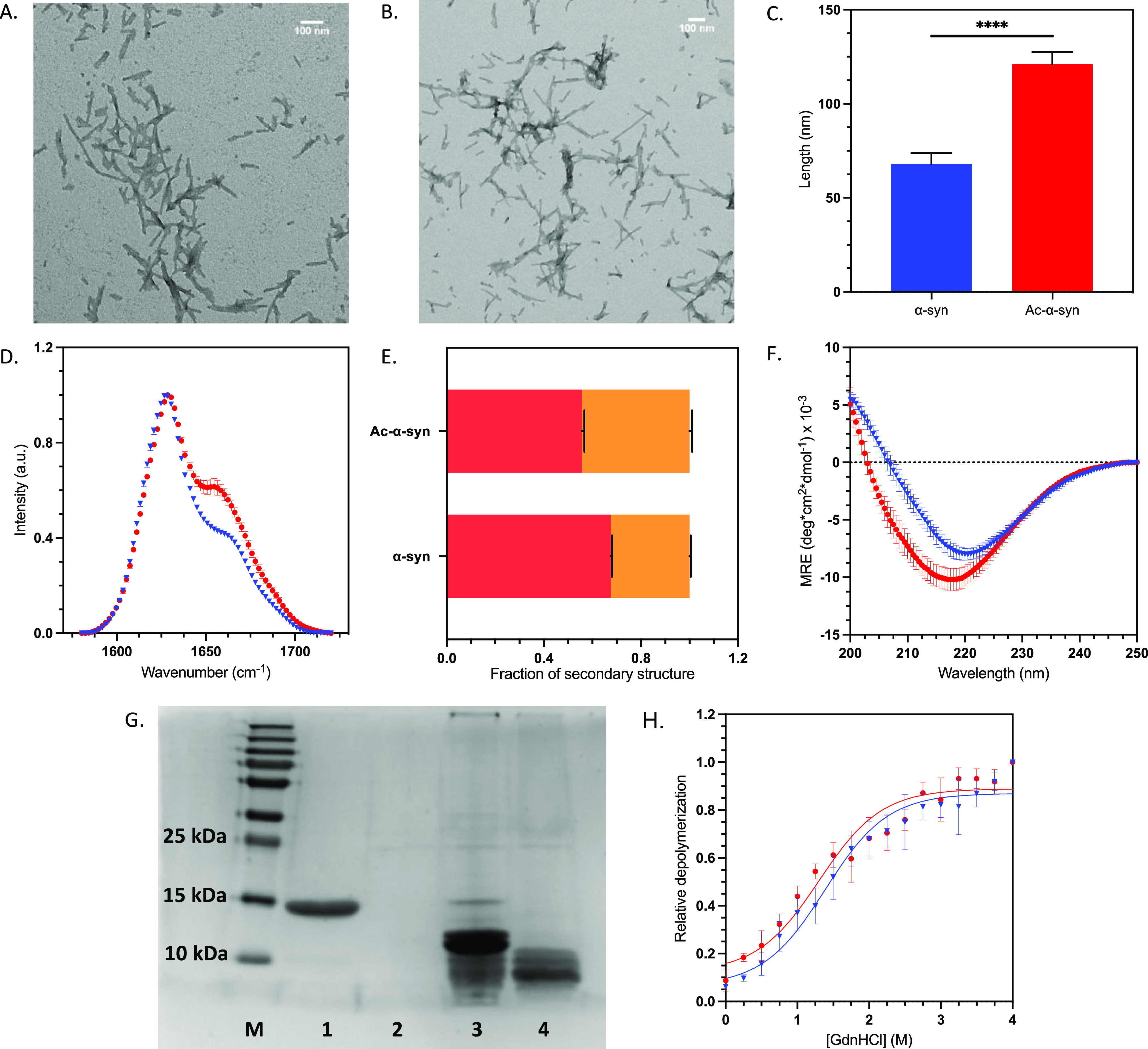
N-terminal acetylation alters the morphology and secondary structure, but not the stability, of α-synuclein fibrils. (A, B) TEM images of F1 α-synuclein fibrils at 14.5 k magnification non-acetylated (A) and acetylated α-synuclein (B) with scale bars of 100 nm (upper right corners). (C) Average length of preformed fibrils (PFFs) calculated by TEM image analysis. The statistical significance was assessed by an unpaired T-test. (D) Normalized FTIR F1 fibrils: non-acetylated α-synuclein (blue triangle) and acetylated α-synuclein (red dot); error bars represent SEM of *n* = 3. (E) Deconvolution of the FTIR spectra into secondary structural elements. β-sheet shown in red and α-helix/disordered in orange. (F) Far UV CD spectra of F1 fibrils: non-acetylated α-synuclein (blue triangle) and acetylated α-synuclein (red dot); error bars represent SEM of *n* = 3, MRE = mean residue ellipticity. (G) SDS-PAGE gel of proteinase K (PK) digests. Lanes are as follows: (M) PageRuler plus protein ladder, (1) non-acetylated α-synuclein monomer, (2) α-synuclein monomer +0.05 mg/mL PK, (3) non-acetylated α-synuclein fibrils +0.05 mg/mL PK, (4) acetylated α-synuclein fibrils +0.05 mg/mL PK. (H) Depolymerization curve using GdnHCl as a denaturant; the concentration of the soluble fraction was measured to observe fibril depolymerization of non-acetylated α-synuclein (blue triangle) and acetylated α-synuclein (red dot); error bars represent SEM of *n* = 3. Data were normalized to the end-point soluble protein concentration at the highest denaturant concentration.

We first probed the morphology of the fibrils, as this could also impact their seeding capacity. Although TEM images of fibrils from both α-synuclein variants showed similar fibrils (Figure 7A,B), we found that preformed fibrils (PFFs, see Materials and Methods Section) from non-acetylated α-synuclein were significantly shorter than those formed by acetylated α-synuclein (Figure 7C). Using CD and FTIR, we then compared the secondary structure of the fibrils formed by non-acetylated and acetylated α-synuclein (Figure 7D,F). By CD, both non-acetylated and acetylated α-synuclein fibrils appeared to have a primarily β-sheet secondary structure, with a characteristic single minimum around 220 nm. However, the spectrum for acetylated α-synuclein fibrils was broader, suggesting the presence of additional secondary structure elements. These results were consistent with those obtained by FTIR, with both α-synuclein fibrils’ secondary structure being dominated by β-sheet with a peak 1624 cm^–1^, but with acetylated α-synuclein fibrils having a significantly increased level of other secondary structures (α-helix or disordered) indicated by the presence of a peak at 1654 cm^–1^. FTIR deconvolution showed that acetylated α-synuclein fibrils have significantly (*p* < 0.01) more disordered and α-helical content compared to non-acetylated α-synuclein fibrils. Furthermore, acetylated α-synuclein fibrils had significantly (*p* < 0.01) less β-sheet content than was observed in non-acetylated α-synuclein fibrils (Figure 7E). In good agreement with the structural differences that we observed by CD and FTIR, partial digestion with proteinase K (PK) indicated an increase in PK accessibility for the acetylated α-synuclein fibrils as compared to the non-acetylated α-synuclein fibrils (Figure 7G). This finding is in line with the proteinase K digestion patterns previously reported for fibrils formed under slightly different aggregation conditions^51^ and confirms that our acetylated α-synuclein fibrils contain more disordered regions. In that study, while acetylated α-synuclein fibrils were found to have different morphological properties compared to non-acetylated α-synuclein fibrils, the amyloid core structure of acetylated α-synuclein fibrils was reported to be similar to the non-acetylated fibrils.^51^ For non-acetylated α-synuclein fibrils, multiple polymorphic structures have been observed, differing in protofibril interface residues, helical rise and twist, and the number of residues with a defined secondary structure in the fibril core.^53^ There is a structure for N-terminally acetylated α-synuclein fibrils that resembles one of these non-acetylated α-synuclein fibril polymorphs;^54^ however, it is also likely that acetylated α-synuclein fibrils can take multiple structural forms. It would be highly relevant to characterize the structures of these species using high-resolution techniques such as cryo-EM.

The stability of the fibrils was assessed by their response to the denaturant guanidine hydrochloride (GdnHCl) (Figure 7H). The sensitivity of fibrils toward GdnHCl-induced denaturation did not vary significantly between non-acetylated and acetylated α-synuclein fibrils. Both fibrillar species appeared to be highly dynamic and showed dissociation at low concentrations of denaturant, suggesting that under destabilizing conditions, α-synuclein fibrils may release monomeric and oligomeric forms through a mechanism independent of surface-catalyzed fibril amplification.

### N-Terminal Acetylation Does Not Modify the Cytotoxicity of α-Synuclein Fibrils and Fibril Fragments

Aggregates of α-synuclein are toxic to cells and induce cell stress and death.^55−57^ Furthermore, previous studies have shown that acetylated α-synuclein is taken up by neuronal cells in a manner distinct to non-acetylated α-synuclein.^46^ Since we observed different morphologies and secondary structure for acetylated and non-acetylated α-synuclein aggregates (Figure 7), we assessed how N-terminal acetylation of α-synuclein affects the cytotoxicity of its aggregates. As fragmented fibrils have an increased toxicity compared to full length mature fibrils,^58^ we used the sonicated PFFs to induce cellular cytotoxicity. PFFs have been used to induce toxicity in mice models by injecting short fibrils of α-synuclein directly into the striatum and shown to recapitulate the phenotypes and spreading of PD, including loss of dopaminergic neurons and reduction of striatal dopamine terminals and motor behavior defects.^57,58^

**Figure 8:**
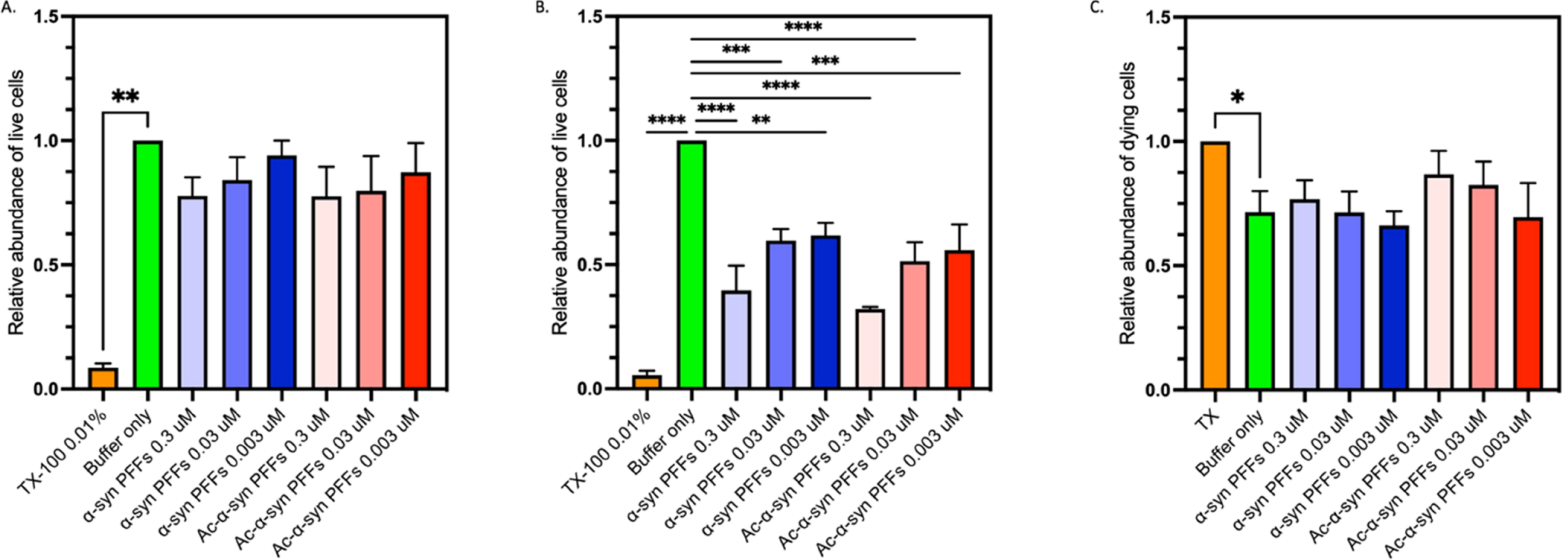
N-terminal acetylation does not modify the cytotoxicity of α-synuclein fibrils and fibril fragments. (A, B) Relative end-point fluorescence intensity of Calcein AM and a live cell dye expressed in arbitrary fluorescence units, in the presence of Triton X-100 (orange) or PBS (green) or when incubated with at 0.3–0.003 μM sonicated fibrils (PFFs) of non-acetylated α-synuclein (blue) and acetylated (Ac) α-synuclein (red) for (A) 24 h and (B) 48 h. (A) All treatments showed significantly different fluorescence levels to Triton X 100 treated cells (*p* < 0.05). However, a significant difference was not found between any other treatments (*p* > 0.05). (B) All treatments, showed significantly different fluorescence levels to buffer only control cells (*p* < 0.05). However, a significant difference was not found between acetylated and non-acetylated preformed sonicated fibril (PFF) treatments (*p* > 0.05). (C) Relative fluorescence intensity of PI expressed in arbitrary fluorescence units, a dead cell detecting dye, in the presence of Triton X-100 (orange) or PBS (green) or when incubated with at 0.3–0.003 μM sonicated fibrils (PFFs) formed from non-acetylated α-synuclein (blue) and acetylated (Ac) α-synuclein (red) for 24 h. * Indicates a *p* value of 0.01; other pairwise comparisons showed no significant differences (*p* > 0.05).

To investigate the cytotoxicity of non-acetylated and acetylated α-synuclein PFFs, we took a two-pronged approach to measure both the impact on live and dying cells with Calcein AM and propidium iodide (PI). Calcein AM is a dye that permeates live cells and is converted to fluorescent calcein by intracellular esterases and therefore acts as a live cell dye for viability measurements.^59^ PI penetrates dead and dying cell membranes, and once inside cells, PI intercalates with DNA to become fluorescent; however, it cannot penetrate live cells and can therefore be used as a cell viability marker.^60,61^ Our results using non-differentiated SH-SY5Y cells indicate no significant difference in the cytotoxicity of the PFFs derived from either non-acetylated or acetylated α-synuclein, as assessed by Calcein AM and PI fluorescence (Figure 8). We also tested the effects of stabilized oligomers from both acetylated and non-acetylated α-synuclein on the viability of SH-SY5Y cells (Figure S4C). Consistent with previous studies,^18^ we found that there was no significant difference in the toxicity induced by the two species.

**Figure 9:**
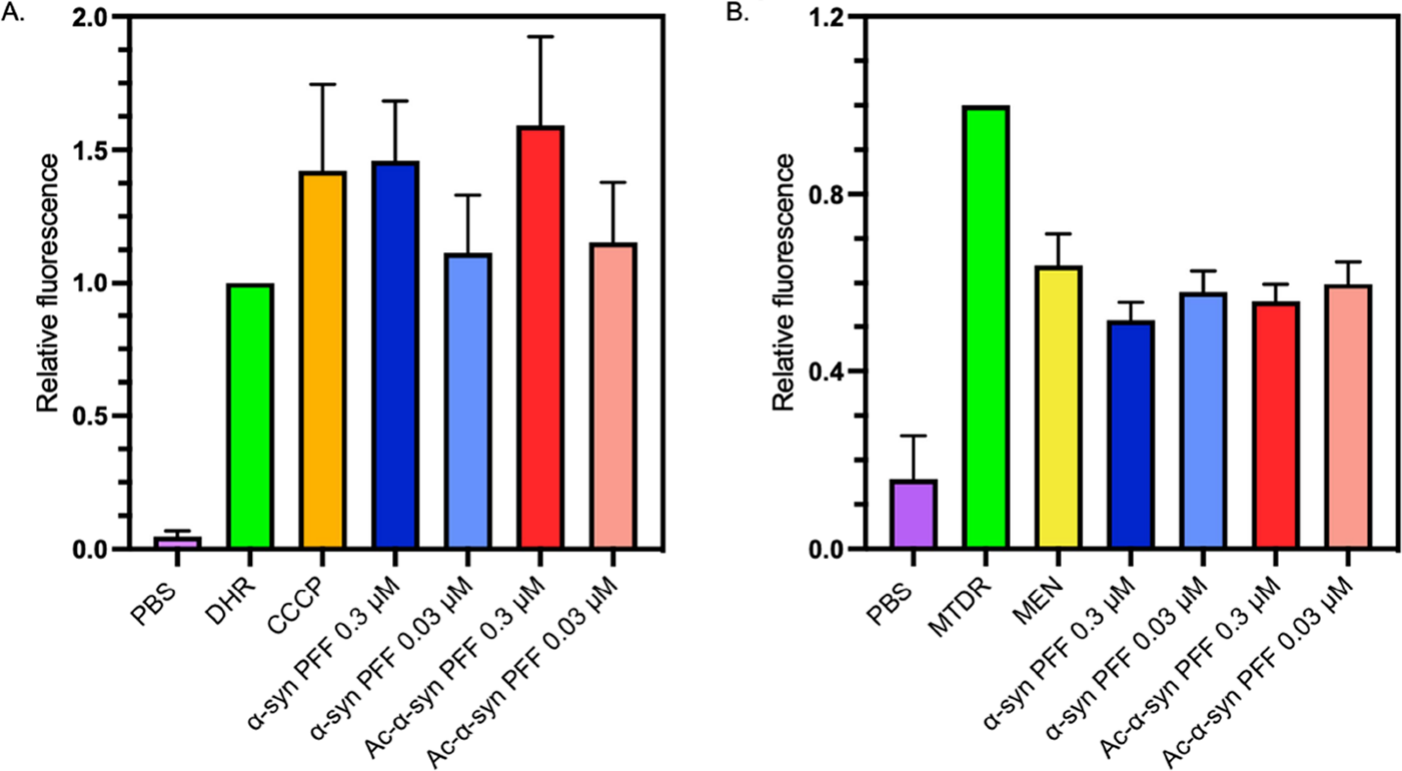
Comparison of the levels of reactive oxygen species (ROS) in the presence of fibrils and fibril fragments formed by non-acetylated and acetylated α-synuclein. (A) Median end-point DHR relative fluorescence intensity. (B) Median end-point MTDR relative fluorescence intensity (A and B) when incubated with 0.3 or 0.03 μM of sonicated fibrils (PFFs) formed from non-acetylated-α-synuclein (blue) and acetylated-α-synuclein (red) for 30 min or with carbonyl cyanide 3-chlorophenylhydrazone (CCCP, orange), an inducer of oxidative stress, or menadione (yellow), a mitochondrial membrane potential decreasing agent for 30 min. Relative fluorescence is expressed in arbitrary fluorescence units. (A) All treatments were significantly different from DHR only (*p* values < 0.03); (B) all treatments were significantly different from MTDR only (*p* values < 0.0001). However, no significant difference was found between acetylated and non-acetylated PFF treatments in A and B (*p* > 0.05). For both, error bars represent SEM for *n* = 3.

When comparing the impact of non-acetylated and acetylated α-synuclein PFFs, we also found that the two variants induced comparable levels of reactive oxygen species (ROS), as measured by dihydrorhodamine (DHR) fluorescence, and disruption of mitochondrial membrane potential, measured by MitoTracker Deep Red (MTDR) fluorescence (Figure 9). Previous studies have found little difference in the cellular localization and distribution of non-acetylated and acetylated α-synuclein,^62^ which may explain the similarity in toxicity we observed for the two species.

## Materials and Methods

### Protein Production

α-Synuclein (UniProt accession code P37840) was produced by transforming *E. coli* with an expression pT7–7 plasmid. N-terminal acetylated α-synuclein was produced by co-transforming *E. coli* with the same plasmid and an expression pACYCduet plasmid encoding a yeast N-terminal acetyltransferase (NatB), provided by Dr. Dan Mulvihill, University of Kent, Canterbury, UK.^24^ Acetylated α-synuclein and non-acetylated α-synuclein were then purified in 20 mM NaPO_4_ pH 6.5 buffer, as described previously.^74^ The presence of N-terminal acetylation was verified by mass spectrometry, and the protein concentrations were determined by absorbance at 275 nm using the extinction coefficient α-synuclein ε = 5600 M^–1^ cm^–1^.^75^ Aliquots were flash-frozen in liquid N_2_ and stored at −80 °C.

### Binding to Lipid Membranes

DMPS small unilamellar vesicles were prepared as described previously,^41^ by cycles of freeze/thaw followed by sonication. DMPS vesicles were added to α-synuclein, and the binding event was measured by CD spectroscopy. CD spectra were obtained on a JASCO J-810 (Easton, USA) using quartz cuvettes with path lengths of 1 mm, by averaging 15 individual spectra recorded between 250 and 200 nm, with a bandwidth of 1 nm, a data pitch of 0.2–0.5 nm, a scanning speed of 50 nm/min, and a response time of 1 s. The relative compositions of the secondary structure were obtained using an online server Bestsel.^65^ To analyze the binding affinity of both acetylated α-synuclein and non-acetylated α-synuclein, the data were fitted using a previous model.^41^

### FTIR Spectroscopy

FTIR spectra were recorded on a Bruker Vertex 70 FTIR (Billerica, USA) on the diamond ATR, with 4 cm resolution and a data range of 800–4000 cm^–1^ wavelengths; the data in the amide peak 1 (1580–1720 cm^–1^) were analyzed. A rubber band baseline correction was applied to the data, before fitting to a Gaussian equation with 4–7 peaks. The area under each peak was integrated to obtain relative compositions of the secondary structure using the following classifications: peaks under 1640 cm^–1^ were assigned to β-sheet structures; peaks from 1640 to 1660 cm^–1^ were assigned to disordered random coils/α-helices, and peaks above 1660 and 1685 cm^–1^ were also assigned to β-sheet structures.

### Solubility Assay

Solubility was measured using a PEG-precipitation assay. In this assay, one measures protein precipitation for increasing concentrations of PEG. The midpoint value (PEG_1/2_) is correlated with the solubility. Monomeric α-synuclein was incubated with increasing concentrations (0–30%) of PEG-6000 at 4 °C for 2 days in 384-well plates. The plates were centrifuged to pellet aggregates, and the supernatant was transferred into a fresh 384-well plate. The monomeric α-synuclein concentration in the supernatant was measured by absorbance. This assay was conducted using the same buffers (and respective pH) specified below for the aggregation reactions and 20 mM NaPO_4_ pH 7.4 also.

### Aggregation Kinetics

To assess the microscopic steps of α-synuclein aggregation, experiments were carried out using different conditions favoring different processes (lipid vesicle induced aggregation, fibril elongation, and surface-catalyzed fibril amplification), all with a range of α-synuclein concentrations from 20–100 μM, and 50 μM ThT^39,41,44^ (Table 1). For the lipid-induced aggregation assay, the concentration of DMPS used was chosen based on previous studies to maintain a low ratio of DMPS/α-synuclein to allow both free α-synuclein and lipid vesicle states to be present, keeping the aggregation rate high.^41^ All assays were set up in triplicate in 96 half-well plates (Corning 3881, non-binding, clear bottomed, Corning, Tewksbury, USA) sealed with aluminum foil, and ThT fluorescence was detected in quiescent conditions on FLUOstar plate readers (BMG Labtech, Aylesbury, UK), with excitation and emission wavelengths of 440 and 480 nm, respectively.

**Table 1:** Conditions Used for the Different Aggregation Assays in This Study

aggregation assay	buffer conditions	temperature
lipid-induced aggregation	20 mM NaPO_4_, pH 6.5, 100 μM DMPS	30 °C
surface-catalyzed fibril amplification	20 mM NaPO_4_, pH 4.8, 50 nM seed	37 °C
fibril elongation	20 mM NaPO_4_, pH 6.5, 2.5–5 μM seed	37 °C

### Aggregation Kinetics under Shaking Conditions

To complement the three-pronged strategy described above and observe the effects of N-terminal acetylation on α-synuclein aggregation in bulk, a shaking method was used in 20 mM NaPO_4_ pH 6.5. α-Synuclein monomers (70 μM) were incubated in Corning 3881 plates in triplicate, 37 °C, with 50 μM ThT and addition of a single glass bead (3 mm diameter) to each well. Aggregation was stimulated by shaking at 200 rpm.

### Fibril Preparation

First generation (F0) fibrils of N-terminal acetylated α-synuclein and non-acetylated α-synuclein were produced by concentrating the monomeric protein to >600 μM and then by incubating for 72–96 h at 40 °C, with 1500 rpm stirring with a Teflon stirrer bar. Fibrils were then sonicated (low power, 50% pulse, 15 s) and left to incubate for a further 24–48 h. The resulting fibrils were pelleted by centrifugation at 15,000 rpm and any remaining monomer removed. Second generation (F1) fibrils were prepared by seeding with 10% concentration of fresh F0 fibrils to monomeric protein concentration. After seeding, the F1 fibrils were sonicated and incubated in the same way as described above. The remaining monomer was again removed by centrifugation, and fibrils were used immediately. Fibril concentrations were measured by dissociating the fibrils in a total solution of 4 M GdnHCl and then measuring the absorbance at 275 nm with the extinction coefficient α-synuclein ε = 5600 M^–1^ cm^–1^.^41^

### Protofibril Isolation

In order to characterize the end-point species of lipid-induced aggregation, aggregates were isolated from the lipid vesicles. N-Lauryl sarcosine sodium salt was added at a 1:1 w/v ratio, and samples were incubated at 37 °C for 1 h. The aggregates were pelleted and washed by ultracentrifugation (120,000 rpm, 1 h, 20 °C) and resuspended in 20 mM NaPO_4_ pH 6.5 three times. The concentration of the monomer in the aggregates was measured as described above for fibrils.

### Preparation of Stabilized Misfolded Oligomers

Stabilized misfolded oligomers were prepared following a previously described method.^52^ Briefly, monomeric α-synuclein at a concentration of ∼850 μM was dialyzed into water, lyophilized, resuspended in PBS, and then incubated at 37 °C for 20–24 h. Large aggregated species were removed by ultracentrifugation (1 h, 288,000 × *g*), and excess monomeric or small oligomeric species were removed by 100 kDa cut-off centrifugation filters. The concentration of monomer in the oligomer samples was measured by absorbance at 275 nm as described for fibril species.

### Proteinase K Digestion of Amyloid Fibrils

Aliquots of the fibril reactions (50 μL) were centrifuged (5 min, 20,000 rcf at room temperature). The supernatant was removed, and the fibrils were resuspended in 50 μL of PBS. To this, proteinase K (0.05 mg/mL final concentration) was added, and the samples were incubated (37 °C, 45 min). The reactions were centrifuged, and the supernatant was removed, 7 M Urea (25 μL) and 4× LDS loading buffer (5 μL) was added, and heated at 95 °C for 5 min. Monomeric α-synuclein (25 μM) was also treated with proteinase K (0.05 mg/mL final concentration) and incubated (37 °C, 45 min) for comparison. Samples were run on a 15% SDS-PAGE gel and stained with Coomassie blue.

### Fibril Stability

To determine the stability of α-synuclein fibrils, a depolymerization assay approach was applied. Fibrils were incubated overnight at 25 °C at increasing concentrations of GdnHCl (0–4 M). Then, samples were centrifuged (15 min, 21,130 × *g*, 20 °C), and the supernatant collected. The concentration of monomeric α-synuclein that had dissociated from fibrils was estimated using a Pierce BCA Protein Assay Kit (ThermoFisher).

### Fibril Morphology

Fibril morphology was analyzed by TEM and AFM. For the TEM analysis, fibrils were diluted to 5–10 μM and incubated on carbon-coated copper grids for 3–4 min before washing with distilled water (dH_2_O) and staining with uranyl acetate (2% w/v) for 2 min and then washed again with dH_2_O. TEM images were taken on a Tecnai G2 80-200kv transmission electron microscope (ThermoScientific, at the Cambridge Advanced Imaging Centre (CAIC), University of Cambridge) with magnifications of 9–14 k. ImageJ was used for length analysis. The same protocol was applied to protofibrils, and they were imaged on a Talos F200X G2 TEM (ThermoScientific, Dept. of Chemistry, University of Cambridge).

AFM samples were prepared following a method previously described (Flagmeier et al.,^44^). Fibrils were diluted to 1 μM in dH_2_O, and 50 μL was deposited onto freshly cleaved mica and incubated for 45 min before washing with 50 μL of dH_2_O. All samples were imaged on a NX10 Atomic Force Microscope (Park Systems, Suwon, South Korea) using non-contact mode. Areas of 4 μm × 4 μm were imaged in 1024 pixels at a speed of 0.3–0.4 Hz. Images were analyzed by SPIP software (Image Metrology, Hørsholm, Denmark) to determine the height and length of aggregates.

### Cell Culture

We used genetically confirmed mycoplasma-free human SH-SY5Y neuroblastoma cells (A.T.C.C., Manassas, USA). The cells were cultured in Dulbecco’s Modified Eagle Medium F-12 Nutrient Mixture + GlutaMax (Gibco, Waltham, USA) with 10% v/v fetal bovine serum (Gibco, Waltham, USA) referred to as SH media. The cells were grown in T-75 cell culture flasks (CELLSTAR, Stonehouse, UK) in a 5.0% CO_2_ humidified atmosphere at 37 °C. Cells were passaged when they were 70–80% confluent.

### Cell Viability Assays

SH-SY5Y cells were plated at a concentration of 10,000 cells per well in a 96-well plate with a lid (3603, Corning) in SH media at a total volume of 200 μL. Surrounding wells were filled with 100 μL sterile PBS to ensure a homogeneous temperature across the plate. The cells were incubated at 37 **°**C for 24 h. The next day, the medium was removed and 200 μL of fresh SH medium with or without treatments was added (0.3, 0.03, or 0.003 μM of the PFFs, PBS, or 0.01% Triton X-100, all treatments were diluted, so that they were diluted 10 times in an appropriate volume of SH media). Each treatment was assessed in sextuplets in a single experiment, and each experiment was repeated at least three times.

For the Calcein AM assay, the SH media was removed from all wells, and cells were washed twice with PBS before a solution of 3 μM Calcein AM in PBS was added and incubated for 30 min at 37 °C. Calcein AM fluorescence was then measured at 485 nm/535 nm excitation/emission. For PI fluorescence assays, the SH media was removed from all wells; a solution of 2.5 μM PI in PBS was then immediately added and incubated for 30 min at 37 **°**C. PI fluorescence was then measured at 530 nm/620 nm excitation/emission. The background level of PI fluorescence was then calculated by removal of PI in PBS solution from all wells, and fresh PBS was then added to wells before a second fluorescence measurement was taken at 530 nm/620 nm. Significant differences in results were identified by one-way ANOVA analysis.

### Cell Toxicity

Flow cytometry experiments were completed following a previously described protocol.^66^ Briefly, cells were resuspended in PBS with no treatments (unstained control) or with 0.1 mM dihydrorhodamine 123 (DHR), and 100 nM MTDR, following no treatment or treatment with ROS-inducing controls 10 μM CCCP, or 10 μM menadione, or with PFFs from non-acetylated or acetylated α-synuclein at 0.3–0.03 μM. CCCP is a proton ionophore that induces oxidation by dissipating the mitochondrial membrane proton gradient,^67^ and menadione also induces oxidative stress in the mitochondria and cytosol and decreases the mitochondrial membrane potential.^68^ Cells were incubated for 30 min at 37 **°**C, then pelleted by centrifugation (250 × *g*, 5 min), and resuspended in PBS and held on ice until analyzed by flow cytometry; 10,000 events were acquired for all samples. Samples were run on a Cytoflex (Beckman), with excitation/emission at 488 and 525/40 nm, respectively, for DHR and excitation/emission at 638 and 660/10 nm for MTDR. DHR is lipophilic and can pass through cell membranes. In cells, DHR is oxidized by free reactive oxygen species (ROS), H_2_O_2_, or peroxynitrite to a fluorescent product.^69^ MTDR is also lipophilic and can permeate cell membranes, where it associates with mitochondria. MTDR is mitochondrial membrane potential sensitive, depolarization decreases fluorescence, while increased potential increases fluorescence.^70^ Significant differences in results were identified by one-way ANOVA analysis.

## Conclusions

The results that we have reported in this work show that N-terminal acetylation retards the aggregation of α-synuclein and alters the secondary structure, length, and morphology of α-synuclein fibrils. We have found in particular that this post-translational modification slows down the aggregation of α-synuclein in the presence of lipid vesicles, while leading to more structured aggregates. Our results also indicate that the N-terminal acetylation increases the reactive flux toward the overall amount of transient misfolded oligomers during the α-synuclein secondary nucleation process. Given that different oligomeric species may give rise to different levels of neurotoxicity^63,64^ it will be important to investigate further the effects of N-terminal acetylation on the lipid-dependent aggregation of α-synuclein for lipid membranes of compositions corresponding to those of synaptic vesicles, endosomes, mitochondria, and other cell membranes.

## Supporting Information Available

Supplementary methods, comparison of lipid binding, comparison of the lipid-induced aggregation, analysis of fibrillar species, and comparison of the structures of stabilized oligomers (PDF)

## Accession Codes

α-synuclein: UniProt P37840.
