# Supplementary material for: N-Terminal Acetylation of α-Synuclein Slows down Its Aggregation Process and Alters the Morphology of the Resulting Aggregates
Source: Biochemistry. 2022 Aug 9;61(17):1743–56. doi: 10.1021/acs.biochem.2c00104 (PMC9454101; doi:10.1021/acs.biochem.2c00104)
Supplement: Supplementary file 1 — bi2c00104_si_001.pdf [file bi2c00104_si_001.pdf]

Supplementary Information

The N-terminal acetylation of α-synuclein slows down its aggregation process and alters the morphology of the resulting aggregates

Rosie Bell[1], Rebecca J. Thrush[1], Marta Castellana-Cruz[1], Marc Oeller[1], Roxine Staats[1], Aishwarya Nene[1], Patrick Flagmeier[1], Catherine K. Xu[1], Sandeep Satapathy[2], Celine Galvagnion[3], Mark R. Wilson[4], Christopher M. Dobson[1], Janet R. Kumita[5*] and Michele Vendruscolo[1*]

Methods

Fibril production. Previous studies have indicated that second-generation fibrils (F1) produce more homogenous and reproducible results compared to first-generation fibrils (F0)[1–3]. In this study, we thus examined the stability of F1 fibrils. Since we found that freezing and thawing of either F0 or F1 fibrils impacted the stability and structure of the fibrils and fibril progeny, only fresh F0 were used to prepare F1 fibrils, and fresh F1 were used in all experiments.

ThT binding assay. Fibril solutions (150 µl, 10 µM) were incubated with ThT (50 µM) for 43 min. The solutions were placed in a cuvette of pathlength 1 cm and fluorescence measurements were recorded on a Cary Eclipse Fluorescence Spectrophotometer (Agilent, USA). Fluorescence emission spectra were recorded at room temperature between 455 to 600 nm, using an excitation of 446 nm and averaging over 10 scans. Background spectra of ThT in the relevant buffer solutions were recorded and subtracted from the fibril sample spectra.

Kinetic analysis of the early time points of lipid induced aggregation. The change in mass concentration of aggregates with time (M(t)) was fitted to a two-step heterogeneous nucleation equation[4,5]

$$M(t) = \frac{Km \cdot k_n k_+ \cdot bt^2 \cdot m(0)^{n+1}}{2(Km + m(0))} \quad \text{Eq. 1}$$

where $m(0)$ is the free monomer concentration at time t = 0, k_+ is the elongation rate constant of fibrils from lipid vesicles, k_n is the heterogeneous primary nucleation rate constant, n is the reaction order of the heterogeneous nucleation reaction relative to the free monomer m. b is the total mass concentration of the protein bound to the lipid at 100% coverage ($b = \frac{[DMPS]}{L}$ with L the stoichiometry). K_M is the Michaelis constant, which was fixed at 125 µM, as determined previously[4,6].

The total amount of α-syn converted to fibrillar species in the aggregation reaction was calculated as done previously [4]. Samples were ultracentrifuged (90 k rpm, 30 min, RT) and the concentration of free monomer was calculated by measuring the absorbance of the samples at 275nm and 410 nm. Using the extinction coefficients for ThT as $\varepsilon_{410} = 3200\ M^{-1}\ cm^{-1}$, $\varepsilon_{275} = 22725\ M^{-1}\ cm^{-1}$, and for α-syn $\varepsilon_{275} = 5600\ M^{-1}\ cm^{-1}$ [4,7].

$$[ThT] = \frac{Abs_{410}}{\varepsilon_{ThT,410}} \quad \text{Eq. 2}$$

$$[\alpha - syn]_{monomer} = \frac{Abs_{275} - [ThT]\varepsilon_{ThT,s75}}{\varepsilon_{\alpha-syn,275}} \quad \text{Eq. 3}$$

Kinetic analysis of seeded α-synuclein aggregation. Under conditions that favour elongation of fibril species and other process can be excluded (µM seed concentrations at pH 6.5), the initial rate of fibril elongation rate can be calculated as described previously[6,8–10].

$$\left.\frac{dM(t)}{dt}\right|_{t=0} = 2k_+ \mathrm{P}(0)m(0) \quad \text{Eq. 4}$$

where $M(t)$ is the fibril mass concentration at time t, $P(0)$ is the initial number concentration of fibrils, $m(0)$ is the initial monomer concentration, and k_+ is the rate of fibril elongation. Under conditions that favour surface-catalysed fibril amplification (nM seed concentration at acidic pH), the rate of fibril amplification determined using equations described previously[8]

$$\frac{M(t)}{m_{tot}} = 1 - \frac{1}{\left[1 + \frac{a}{c}e^{xt}\right]^c}, \quad a = \frac{\lambda^2}{2\kappa^2}, \quad c = \sqrt{\frac{2}{n_2(n_2+1)}}, \quad \lambda = \sqrt{2k_+k_n m_{tot}^{n_c}},$$

$$\kappa$$

$$= \sqrt{2k_+ k_2 m_{tot}^{n_2+1}} \qquad \text{Eq. 5}$$

where m_{tot} is the total concentration of monomers (in this case $m(0) = m_{tot}$), a and κ and c as fitting parameters, k_n and k_2 are the rate constants for primary and secondary nucleation, and n_c and n_2 are the reaction orders of primary and secondary nucleation, respectively[6,8,9]. κ and λ represent combinations of the effective primary and secondary rate constants, respectively[8,11].

The flux towards oligomeric species, $\phi(t)$, in the reaction was also predicted using equations described previously[8,11]

$$\phi(t) = \frac{1}{r_+} \cdot \left[\frac{m(0)}{m(t)} \cdot \frac{d^2M}{dt^2} + \frac{1}{m(0)} \left(\frac{m(0)}{m(t)} \cdot \frac{dM(t)}{dt}\right)^2\right] \qquad \text{Eq. 6}$$

where $r_+ = 2k_+m(0)$ is the rate of elongation calculated from elongation-favouring conditions as described above[8].

Analysis of lipid binding

The data were analysed using Bayesian inference to obtain values for K_D and stoichiometry, using equations from[4]. Briefly, we took the CD signal at 222 nm as a measure of helical propensity, and assumed the observed data can be described as

$$CD_{obs} = x_B CD_B + x_F CD_F \qquad \text{Eq. 7}$$

where x_B and x_F are the fractions of α-syn bound to the membrane and free in solution, respectively, and CD_B and CD_F are the CD signals of the bound and free form of α-syn, respectively.

From this, x_B can be calculated by assuming $x_B + x_F = 1$, and CD_F and CD_B are equal to the signal of α-syn monomer in buffer alone, or with saturating concentrations of SUVs respectively

$$x_B = \frac{CD_{obs} - CD_F}{CD_B - CD_F} \qquad \text{Eq. 8}$$

From this, we can fit the data to obtain values for K_D and stoichiometry of number of DMPS molecules interacting with a molecule of α-syn (L)[4]

$$x_B = \frac{\left([\alpha-syn]+\frac{[DMPS]}{L}+K_D\right)-\sqrt{\left([\alpha-syn]+\frac{DMPS}{L}+K_D\right)^2-\frac{4[DMPS][\alpha-syn]}{L}}}{2[\alpha-syn]} \qquad \text{Eq. 8}$$

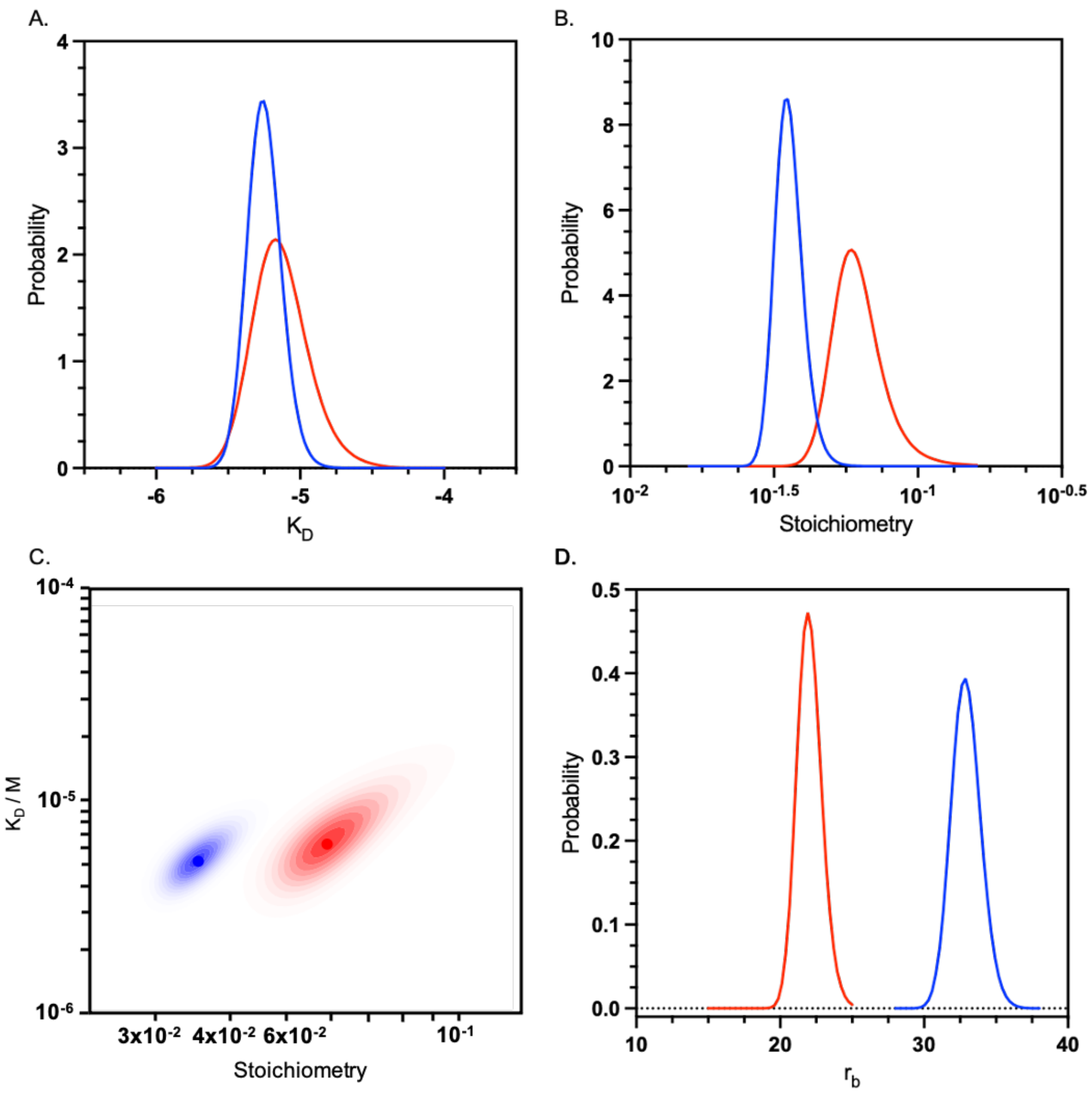

Figure S1. Comparison of lipid binding of the non-acetylated and N-terminal acetylated α-synuclein. The binding of α-synuclein to DMPS vesicles was measured by observing the changes in secondary structure using far UV CD. Bayesian inference was used to obtain values for K_D and L. (**A**) Log(K_d) probability plots of the non-acetylated (blue) and acetylated (red) α-synuclein. (**B**) Log(Stoichiometry), of DMPS molecules per α-synuclein monomer, probability plots of the non-acetylated (blue) and acetylated (red) α-synuclein. (**C**) Log(K_d) versus stoichiometry plots of the non-acetylated (blue) and acetylated (red) α-synuclein. (**D**) Helicity of fully bound α-synuclein, r_b = mean residue ellipticity at 222 nm x 10^{-3}, 3 mM DMPS.

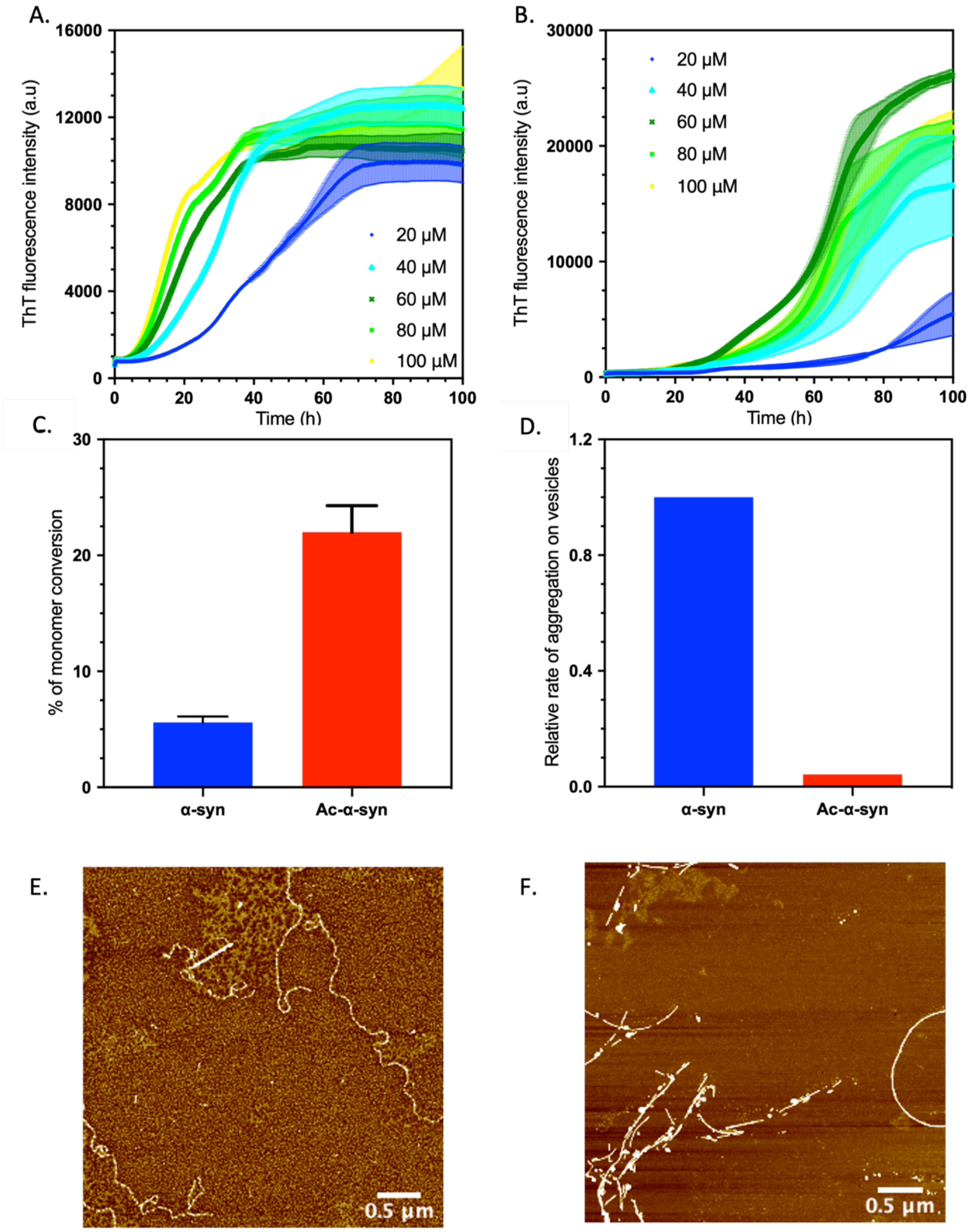

Figure S2. Comparison of the lipid-induced aggregation of the non-acetylated and N-terminal acetylated forms of α-synuclein. (A,B) Representative traces of lipid-induced aggregation assay of non-acetylated α-synuclein (**A**) and acetylated α-synuclein (**B**); for both forms, increasing initial concentrations of α-synuclein monomers added to the reaction are shown: 20 μM (dark blue), 40 μM (light blue), 60 μM (green), 80 μM (light green), and 100 μM (yellow). (**C**) The amount of monomeric α-synuclein converted to aggregate in the lipid-induced nucleation assay was calculated by measuring the concentration of monomeric species

left at the end of the assay. **(D)** One-step nucleation curves fitted to the early timepoints of lipid-induced aggregation data by the fitting program Amylofit [5] using Eq. 1 from supplementary methods to calculate values for k_nk_+. k_+ is the elongation rate constant of fibrils from lipid vesicles, k_n is the heterogeneous nucleation rate constant, n is the reaction order of the heterogeneous nucleation reaction relative to the free monomer. Non-acetylated α-synuclein has a $k_nk_+ = 1.30 \times 10^{-5}\ M^{-(n+1)}\ s^{-2} \pm 2.24 \times 10^{-6}$ and $n = 0.712 \pm 0.012$. and for acetylated α-synuclein. Acetylated α-synuclein $k_nk_+ = 5.52 \times 10^{-7}\ M^{-(n+1)}\ s^{-2} \pm 1.00 \times 10^{-8}$ and $n = 0.712$. **(E,F)** AFM images at the end point of lipid-induced aggregation reaction (**Figure 4C,D**), for non-acetylated α-synuclein (**E**) and acetylated α-synuclein (**F**).

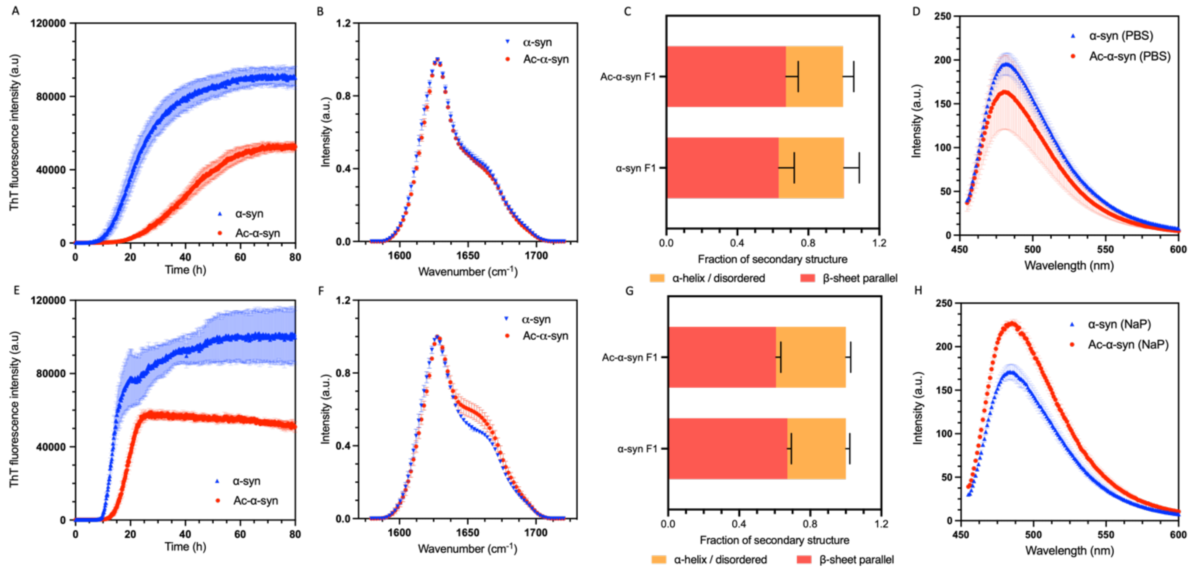

Figure S3. Analysis of fibrillar species obtained from an in vitro aggregation reaction under shaking conditions. Aggregation of α-synuclein was stimulated by shaking and measured by ThT fluorescence intensity. Non-acetylated α-synuclein (blue) and acetylated α-synuclein (red) aggregation was performed in 20 mM NaPO4 pH 6.5 (**A**) and in PBS pH 7.4. The secondary structure of the fibrils from the shaking induced aggregation were then analysed by FTIR and the spectra deconvoluted to reveal secondary structural characteristics of the aggregates. (**B**) FTIR spectra of non-acetylated α-synuclein (blue) and acetylated α-synuclein (red) fibrils formed from the bulk kinetics reaction in 20 mM NaPO4 pH 6.5 buffer, (**C**) deconvolution of the FTIR spectra into secondary structural elements, (**D**) fluorescence spectra of the ThT bound fibrils. (**F**) FTIR spectra of non-acetylated α-synuclein (blue) and acetylated α-synuclein (red) fibrils formed from the bulk kinetics reaction in PBS pH 7.4, (**G**)

deconvolution of the FTIR spectra into secondary structural elements (**H**) fluorescence spectra of the ThT bound fibrils. For A - H error bars represent SEM of 3 repeats.

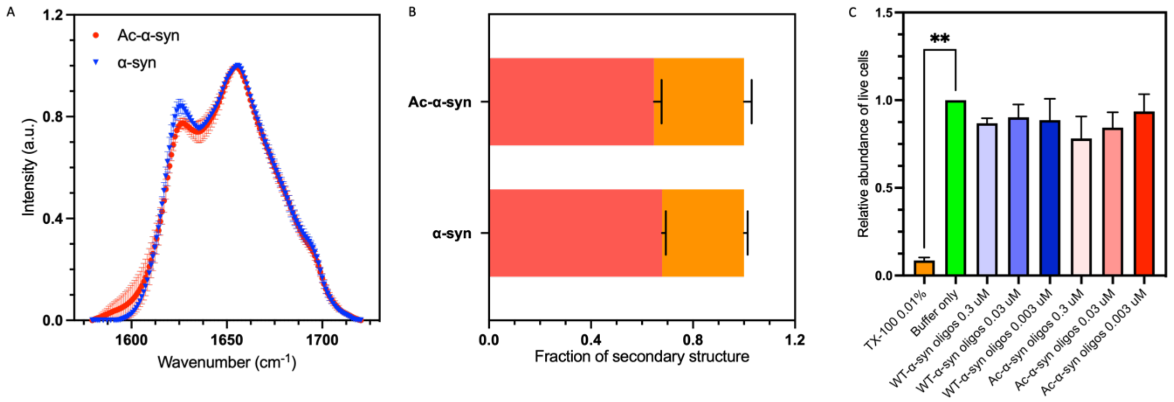

Figure S4. Comparison of the structures of stabilised oligomers of non-acetylated α-synuclein and acetylated α-synuclein. (**A**) FTIR spectra of non-acetylated α-synuclein (blue triangle) and acetylated α-synuclein (red dot); error bars represent SEM of n = 3. (**B**) deconvolution of the FTIR spectra into secondary structural elements (*β*-sheet shown in red and *α*-helix/disordered shown in orange); error bars represent SEM of n = 3. (**C**) Relative end-point fluorescence intensity of Calcein AM, a live cell dye expressed in arbitrary fluorescence units, in untreated cells (dark green) or in the presence of Triton X-100 (orange), PBS (green) or when incubated with at 0.3 µM - 0.003 µM stabilised oligomers consisting of non-acetylated α-synuclein (blue) and acetylated (Ac) α-synuclein (red). No significant difference was found between acetylated and non-acetylated oligomer treatments at equivalent concentrations ($p > 0.05$). Error bars represent SEM for n = 3.

Supplementary References

(1) Aprile, F. A., Arosio, P., Fusco, G., Chen, S. W., Kumita, J. R., Dhulesia, A., Tortora, P., Knowles, T. P. J., Vendruscolo, M., Dobson, C. M., and Cremades, N. (2017) Inhibition of α-Synuclein Fibril Elongation by Hsp70 Is Governed by a Kinetic Binding Competition between α-Synuclein Species. *Biochemistry 56*, 1177–1180.
(2) Chen, S. W., and Cremades, N. (2018) Preparation of α-Synuclein Amyloid Assemblies for Toxicity Experiments. *Amyloid Proteins. Methods Mol. Biol. 1779*.
(3) Polinski, N. K., Volpicelli-Daley, L. A., Sortwell, C. E., Luk, K. C., Cremades, N., Gottler, L. M., Froula, J., Duffy, M. F., Lee, V. M. Y., Martinez, T. N., and Dave, K. D. (2018) Best practices for generating and using alpha-synuclein pre-formed fibrils to model Parkinson's disease in rodents. *J. Parkinsons. Dis. 8*, 303–322.
(4) Galvagnion, C., Buell, A. K., Meisl, G., Michaels, T. C. T., Vendruscolo, M., Knowles, T. P. J., and Dobson, C. M. (2015) Lipid vesicles trigger α-synuclein aggregation by stimulating primary nucleation. *Nat. Chem. Biol. 11*, 229–234.
(5) Meisl, G., Kirkegaard, J. B., Arosio, P., Michaels, T. C. T., Vendruscolo, M., Dobson, C. M., Linse, S., and Knowles, T. P. J. (2016) Molecular mechanisms of protein aggregation from global fitting of kinetic models. *Nat. Protoc. 11*, 252–272.
(6) Flagmeier, P., Meisl, G., Vendruscolo, M., Knowles, T. P. J., Dobson, C. M., Buell, A. K., and Galvagnion, C. (2016) Mutations associated with familial Parkinson's disease alter the initiation and amplification steps of α-synuclein aggregation. *Proc. Natl. Acad. Sci. 113*, 10328–10333.
(7) Weinreb, P. H., Zhen, W., Poon, A. W., Conway, K. A., and Lansbury, P. T. (1996) NACP, a protein implicated in Alzheimer's disease and learning, is natively unfolded. *Biochemistry 35*, 13709–13715.
(8) Staats, R., Michaels, T. C. T., Flagmeier, P., Chia, S., Horne, R. I., Habchi, J., Linse, S., Knowles, T. P. J., Dobson, C. M., and Vendruscolo, M. (2020) Screening of small molecules using the inhibition of oligomer formation in α-synuclein aggregation as a selection parameter. *Commun. Chem. 3*, 1–9.
(9) Gaspar, R., Meisl, G., Buell, A. K., Young, L., Kaminski, C. F., Knowles, T. P. J., Sparr, E., and Linse, S. (2017) Secondary nucleation of monomers on fibril surface dominates α-synuclein aggregation and provides autocatalytic amyloid amplification. *Q. Rev. Biophys. 50*.
(10) Buell, A. K., Galvagnion, C., Gaspar, R., Sparr, E., Vendruscolo, M., Knowles, T. P. J., Linse, S., and Dobson, C. M. (2014) Solution conditions determine the relative importance of

nucleation and growth processes in α-synuclein aggregation. *Proc. Natl. Acad. Sci. U. S. A. 111*, 7671–7676.

(11) Michaels, T. C. T., Cohen, S. I. A., Vendruscolo, M., Dobson, C. M., and Knowles, T. P. J. (2016) Hamiltonian Dynamics of Protein Filament Formation. *Phys. Rev. Lett. 116*, 038101.
